# High Return-to-Golf Rates and Satisfaction After Shoulder Arthroscopy and Arthroplasty: A Comparative Study

**DOI:** 10.7759/cureus.109355

**Published:** 2026-05-21

**Authors:** Leonard Stokes, Vidhur Polam, Yousy M Sauvanell, Tammy Hoffman, Samer Al-Humadi, Patrick J Denard

**Affiliations:** 1 Orthopedics, Oregon Shoulder Institute, Medford, USA; 2 Orthopedics, Kaleida Health Olean General Hospital, Olean, USA

**Keywords:** arthroscopy and arthroplasty, arthroscopy and sports related injuries, golf, retrospective comparative study, total shoulder arthroplasty

## Abstract

Background and objective

Golf is a popular recreational activity among older adults, many of whom develop shoulder pathology requiring surgical intervention. Despite increasing interest in return-to-sport outcomes, comparative data on return to golf following shoulder arthroscopy versus shoulder arthroplasty remain limited. This study aims to compare return-to-golf outcomes, patient satisfaction, and functional recovery between patients undergoing shoulder arthroscopy and those undergoing shoulder arthroplasty. This is a retrospective cohort study and represents level III evidence.

Methods

A retrospective comparative study was performed on 71 recreational golfers, including 46 (64.8%) who underwent arthroscopy and 25 (35.2%) who underwent arthroplasty. Pre- and postoperative assessments included range of motion (ROM) and patient-reported outcome measures (PROMs). The PROMs included the American Shoulder and Elbow Surgeons (ASES) score, visual analog scale (VAS), and subjective shoulder value (SSV). Golf-specific outcomes included return timelines, satisfaction, and performance. Paired t-tests assessed within-group changes, and independent t-tests evaluated between-group differences.

Results

Both groups demonstrated significant improvements in ASES, VAS, and SSV scores (all p < 0.001). Forward flexion and external rotation improved more in the arthroplasty group (p = 0.001 and p = 0.030), while internal rotation did not significantly improve in either group. The mean time to resume putting, chipping, and driving was 5.8 ± 3.1 months, 6.2 ± 3.0 months, and 7.4 ± 2.9 months, respectively, with no significant differences between groups (putting, p = 0.890; chipping, p = 0.894; driving, p = 0.990). At 12 months, 57 of 71 patients (80.3%) had returned to golf (p = 0.970), and 65 of 71 patients (91.5%) reported being satisfied or very satisfied.

Conclusion

Recreational golfers undergoing either shoulder arthroscopy or arthroplasty experience significant functional improvements and high return-to-golf rates. Surgical technique was not associated with differences in return timelines, satisfaction, or golf-specific performance. These findings may guide patient expectations and support shared decision-making in surgical planning.

## Introduction

Golf is one of the most widely played recreational sports in the United States, with over 25 million active participants annually [1]. Although often perceived as low-impact, the golf swing involves complex, repetitive upper-extremity motion that places substantial stress on the rotator cuff, glenohumeral joint, and surrounding musculature [2]. Consequently, shoulder pain is common among golfers, with rotator cuff tears and glenohumeral osteoarthritis being two of the most frequently encountered pathologies [3]. For many recreational athletes, returning to golf serves as a key benchmark of recovery and functional success following treatment.

Shoulder dysfunction can significantly affect golfers, with pain, reduced range of motion (ROM), and diminished swing power impairing both performance and quality of life. While conservative treatments such as physical therapy, activity modification, and corticosteroid injections may provide temporary relief, many patients ultimately require surgical intervention [4]. Common procedures include arthroscopic rotator cuff repair (ARCR), total shoulder arthroplasty (TSA), and reverse shoulder arthroplasty (RSA). The ARCR is typically indicated for reparable rotator cuff tears and has demonstrated favorable outcomes, including return-to-sport rates exceeding 70% in recreational athletes [5,6]. Total shoulder arthroplasty is generally indicated for advanced glenohumeral osteoarthritis, while RSA is reserved for cuff tear arthropathy or irreparable rotator cuff tears. Both procedures offer excellent pain relief and functional restoration, with modern studies reporting high return-to-golf rates, particularly among older individuals [6,7,8].

Despite the increasing prevalence of shoulder surgery among aging athletes, few studies have directly compared sport-specific outcomes between shoulder arthroscopy and arthroplasty, particularly with respect to return-to-golf timelines, performance, and satisfaction. This study compares return to golf timelines, clinical outcomes, and patient-reported satisfaction among recreational golfers who underwent shoulder arthroscopy, primarily arthroscopic rotator cuff repair, and those who underwent shoulder arthroplasty, including both anatomic and reverse total shoulder arthroplasty. While arthroscopy and arthroplasty address distinct pathologies, many older golfers present with overlapping symptoms. Comparing return-to-golf rates provides procedure-agnostic benchmarks for patient counseling, especially in borderline cases where either might be considered. We hypothesized that both procedures would yield similar return-to-golf rates and functional recovery, despite their differing surgical indications and techniques.

## Materials and methods

This retrospective comparative study included patients who underwent shoulder arthroscopy or shoulder arthroplasty between 2014 and 2022 at a single institution and who regularly played recreational golf before surgery. This study was determined to be exempt from Institutional Review Board (IRB) oversight by the Southern Oregon IRB (Medford, OR, USA) under 45 CFR 46.104(d)(2), with limited IRB review conducted to confirm minimal risk to participants. Inclusion criteria were (1) completion of both preoperative and postoperative clinical assessments, (2) completion of a documented return-to-golf survey, and (3) a minimum of one year of clinical follow-up. Patients who underwent revision surgery or had incomplete data were excluded. The arthroscopy group primarily consisted of patients treated with arthroscopic rotator cuff repair, though a small number underwent isolated subacromial decompression or debridement. The arthroplasty group included both anatomic TSA and RSA.

Collected variables included demographic characteristics; shoulder range of motion (ROM), i.e., forward flexion (FF), external rotation (ER), and internal rotation (IR); and patient-reported outcome measures (PROMs) per the American Shoulder and Elbow Surgeons (ASES) score [9], visual analog scale (VAS) for pain, and subjective shoulder value (SSV) [10]. Return-to-golf data included time to resume play, satisfaction with performance, and return to specific golf activities (putting, chipping, and driving).

Postoperative ROM measurements and patient-reported outcome measures were obtained at the most recent clinical follow-up visit, with a minimum of 12 months postoperatively. Return-to-golf data were collected via a survey administered at or beyond the 12-month postoperative mark. Paired t-tests were used to evaluate pre- versus postoperative changes within each surgical group. Independent t-tests were used to compare postoperative values and return-to-golf timelines between the arthroscopy and arthroplasty cohorts. A p-value of <0.05 was considered statistically significant.

## Results

Demographic characteristics

A total of 71 patients met the inclusion criteria: 46 (64.8%) who underwent shoulder arthroscopy and 25 (35.2%) who underwent shoulder arthroplasty. The arthroscopy group primarily consisted of patients who underwent arthroscopic rotator cuff repair (40 [87.0%]), with a smaller number treated with arthroscopic capsular release (three, 6.5%) or debridement (three, 6.5%). The arthroplasty group included patients who underwent either anatomic TSA (12, 48.0%) or RSA (13, 52.0%). The average age was 60.3 ± 8.7 years in the arthroscopy group compared to 69.4 ± 4.8 years in the arthroplasty group (p < 0.001). The majority of patients in both groups were male (36 of 46 (78.3%) in the arthroscopy group and 21 of 25 (84.0%) in the arthroplasty group; p = 0.788), with similar BMI and follow-up durations (Table 1).

**Table 1:** Demographics by procedure type Values are presented as n (%). Percentages for the number of patients are based on the total cohort (n = 71), while procedure type and sex percentages are calculated within each surgical group. ACR: Arthroscopic capsular release; ARCR: Arthroscopic rotator cuff repair; RSA: Reverse shoulder arthroplasty; TSA: Total shoulder arthroplasty

Variable	Arthroscopy, n (%)	Arthroplasty, n (%)
Number of patients	46 (64.8%)	25 (35.2%)
Procedure type	ARCR = 40 (87.0%), ACR = 3 (6.5%), debridement = 3 (6.5%)	TSA = 12 (48.0%), RSA = 13 (52.0%)
Age (mean ± SD)	60.3 ± 8.7	69.4 ± 4.8
Male, n (%)	36 (78.3%)	21 (84.0%)
BMI (mean ± SD)	29.1 ± 4.3	28.7 ± 3.9
Follow-up time (months, mean ± SD)	18.2 ± 6.4	19.0 ± 5.8

Range of motion outcomes

Both groups demonstrated significant postoperative improvements in forward flexion and external rotation. In the arthroscopy group, forward flexion improved from 118° to 157°, representing a mean increase of 39° (95% CI, 28-49; p < 0.001). In the arthroplasty group, forward flexion improved from 92° to 162°, with a mean increase of 70° (95% CI, 56-84; p <0.001). External rotation improved from 33° to 72° in the arthroscopy group and from 25° to 76° in the arthroplasty group, corresponding to mean increases of 39° (95% CI, 33-44) and 51° (95% CI, 42-61), respectively (both p < 0.001). Internal rotation remained unchanged in both groups (arthroscopy Δ = 0, 95% CI, -1 to 1; p = 0.534; arthroplasty Δ = 0, 95% CI, -2 to 2; p = 0.824). Between-group analysis demonstrated greater improvements in forward flexion and external rotation in the arthroplasty group compared to the arthroscopy group (p = 0.001 and p = 0.030, respectively), while internal rotation was similar between groups (p = 0.639; Table 2).

**Table 2:** Comparison of shoulder range of motion before and after surgery Values for forward flexion and external rotation are reported in degrees (°). Internal rotation spinal level are coded as T10, 10; T12, 12; L2, 14; L4, 16; S1, 18; hip, 20. ROM: Range of motion

ROM measure	Arthroscopy (n = 46)		Arthroplasty (n = 25)		p-value (between groups)
	Preoperative	Postoperative	Preoperative	Postoperative	
Forward flexion	118°	157°	92°	162°	0.001
External rotation	33°	72°	25°	76°	0.030
Internal rotation	L4	L4	L4	L4	0.639

Patient-reported outcomes

The PROMs measured after a minimum of one-year follow-up improved significantly in both groups (Table 3). In the arthroscopy group, the ASES score improved by 39.4 points (95% CI, 34.3-44.4; p < 0.001), VAS improved by 4.4 points (95% CI, -5.0 to -3.8; p < 0.001), and SSV improved by 42.1 points (95% CI, 35.1-49.0; p < 0.001). In the arthroplasty group, the ASES score improved by 42.7 points (95% CI, 35.5-49.9; p < 0.001), VAS improved by 4.6 points (95% CI, -5.4 to -3.9; p < 0.001), and SSV improved by 44.9 points (95% CI, 35.1-54.7; p < 0.001). No statistically significant differences were observed between groups for postoperative PROMs (Table 3).

**Table 3:** Changes in PROMs following surgery ASES: American Shoulder and Elbow Surgeons; PROMs: Patient-reported outcome measures; SSV: Subjective shoulder value; VAS: Visual analog scale; Δ: Change

PROMs	Arthroscopy (n = 46)		Arthroplasty (n = 25)		p-value (between groups)
	Mean change (Δ)	95% CI	Mean change (Δ)	95% CI	
ASES score	39.4	34.3-44.4	42.7	35.5-49.9	0.560
VAS	-4.4	-5.0 to -3.8	-4.6	-5.4 to -3.9	0.773
SSV	42.1	35.1-49.0	44.9	35.1-54.7	0.668

Return-to-golf outcomes, satisfaction, and performance

Return to golf within 12 months of surgery was achieved by 37 of 46 patients (80.4%) in the arthroscopy group and 20 of 25 patients (80.0%) in the arthroplasty group (p = 0.970). The mean time to resume putting, chipping, and driving in the overall cohort was 5.8 ± 3.1 months, 6.2 ± 3.0 months, and 7.4 ± 2.9 months, respectively, with no significant differences between groups (putting: p = 0.890; chipping: p = 0.894; driving: p = 0.990; Table 4). Among the 14 of 71 patients (19.7%) who had not returned to golf by 12 months, reasons for non-return were not systematically captured by the survey instrument and represent a limitation of this study.

**Table 4:** Return-to-golf timelines after shoulder surgery

Measure	Total cohort (n = 71)	Arthroscopy (n = 46)	Arthroplasty (n = 25)	p-value
Time to putt (mean ± SD)	5.84 ± 3.07	5.88 ± 3.26	5.78 ± 2.71	0.890
Time to chip (mean ± SD)	6.15 ± 3.04	6.19 ± 3.05	6.08 ± 3.04	0.894
Time to drive (mean ± SD	7.38 ± 2.86	7.38 ± 3.09	7.38 ± 2.42	0.990

Patient-reported satisfaction with return to golf was high across both cohorts, with 65 of 71 patients (91.5%) reporting being satisfied or very satisfied. Among patients, 25 of 46 (54.3%) in the arthroscopy group and 18 of 25 (72.0%) in the arthroplasty group reported being very satisfied (Table 5). With respect to perceived golf performance, among patients, improvement was reported by seven of 46 (15.2%) arthroscopy patients and nine of 25 (36.0%) arthroplasty patients. Most patients reported no change in performance (29 of 46 (63.0%) vs. 11 of 25 (44.0%)), while worsening performance was reported by seven of 46 (15.2%) and five of 25 (20.0%) patients, respectively (Table 5).

**Table 5:** Golf-specific outcomes regarding satisfaction and performance The values are presented as n (%). 'Not applicable' indicates a response not provided.

Outcome		Total cohort (n = 71)	Arthroscopy (n = 46)	Arthroplasty (n = 25)
Satisfaction, n (%)	Very satisfied	43 (60.6%)	25 (54.3%)	18 (72.0%)
	Satisfied	22 (31.0%)	16 (34.8%)	6 (24.0%)
	Neutral	3 (4.2%)	3 (6.5%)	0 (0.0%)
	Not applicable	2 (2.8%)	1 (2.2%)	1 (4.0%)
	Very dissatisfied	1 (1.4%)	1 (2.2%)	0 (0.0%)
Performance change, n (%)	Stayed the same	40 (56.3%)	29 (63.0%)	11 (44.0%)
	Worsened	12 (16.9%)	7 (15.2%)	5 (20.0%)
	Improved	16 (22.5%)	7 (15.2%)	9 (36.0%)
	Not applicable	3 (4.2%)	3 (6.5%)	0 (0.0%)

## Discussion

This study addresses a key gap in the literature by directly comparing return-to-golf outcomes between shoulder arthroscopy and shoulder arthroplasty in recreational golfers. We found that 57 of 71 patients (80.3%) successfully returned to golf within 12 months, with an overall satisfaction rate of 65 of 71 patients (91.5%) reporting being satisfied or very satisfied and similar timelines for returning to putting (5.8 ± 3.1 months), chipping (6.2 ± 3.0 months), and driving (7.4 ± 2.9 months). These findings suggest that both procedures can support meaningful return to golf-specific activities, although the comparative interpretation is limited by sample size and the heterogeneity of underlying surgical indications.

The return-to-golf rates and timelines we found in our study are consistent with prior studies evaluating outcomes after shoulder surgery. Tramer et al. reported that most patients undergoing ARCR resumed golf within six months, with high satisfaction and preserved performance, which closely aligns with our findings of 57 of 71 patients (80.3%) returning to golf and skill-specific resumption by approximately six to seven months [11]. Williamson et al. emphasized the role of swing phase modifications and structured rehabilitation in optimizing return-to-play outcomes following ARCR, a theme supported by our observed satisfaction rate of 41 out of 46 (89.1%) arthroscopy patients reporting being satisfied or very satisfied [12]. Similarly, prior studies of shoulder arthroplasty, including both TSA and RSA procedures, have demonstrated high rates of return to golf, though often with swing modifications. Mannava et al. noted that 93.7% of TSA patients returned to play, with a majority of them reporting improved pain control [13]. Salem et al. found that approximately 80% of TSA patients resumed golf with preserved driving distance and swing mechanics [6]. Lansdown et al. reported a 79% return rate following RSA, with the majority of patients returning to play by six months, though many modified their swing [7]. Robinson et al. similarly found RSA return-to-golf rates exceeding 70%, particularly in recreational players [8]. Our findings, with 20 of 25 patients (80.0%) in the arthroplasty group returning to play by 12 months, support these trends and provide a detailed timeline for staged return to specific golf activities.

In addition to return-to-golf metrics, both surgical cohorts experienced significant functional improvements, as evidenced by substantial gains in the ASES, VAS, and SSV scores (all p < 0.001). These improvements mirror the long-term functional outcomes reported by Antoni et al. following ARCR [14] and are comparable to results from Aim et al., who demonstrated high satisfaction and return-to-sport rates following shoulder arthroplasty [15]. Forward flexion and external rotation improved more in the arthroplasty group (p = 0.001 and p = 0.030), consistent with prior studies indicating that TSA may provide greater restoration of overhead motion due to joint resurfacing and pain relief. Similarly, RSA has been associated with improved forward elevation through deltoid compensation enabled by glenosphere medialization and a fixed center of rotation, although limitations in internal rotation are frequently observed [7,8,16]. However, internal rotation did not significantly improve in either cohort, which may reflect persistent subscapularis weakness or altered shoulder mechanics postoperatively, a finding also noted by Takayama et al. in their analysis of anterior cuff fixation strategies [17]. The similarity in PROM gains across arthroscopy and arthroplasty suggests that, despite differing surgical indications and techniques, both procedures yield reliable improvements in pain and function for recreational golfers.

Patient-reported satisfaction with return to golf was high across both cohorts, with 65 of 71 patients (91.5%) reporting being satisfied or very satisfied, and a greater proportion of arthroplasty patients being very satisfied compared to arthroscopy patients (75.0% vs 54.5%). Of note, the satisfaction rate (65 of 71, 91.5%) exceeded the return-to-golf rate (57 of 71, 80.3%), indicating that several patients who had not yet fully returned to play nonetheless reported satisfaction with their recovery trajectory. Notably, nine of 25 patients (35.0%) in the arthroplasty group and seven of 46 patients (15.2%) in the arthroscopy group reported improved golf performance, while a minority in each group reported worsening performance. These findings suggest that shoulder arthroplasty, despite its more invasive nature, may offer comparable or even superior subjective performance outcomes compared to arthroscopy, likely due to greater pain relief and restoration of overhead mechanics. That said, the numerically higher proportion of arthroplasty patients reporting improved performance (35.0% vs 15.2%) should be interpreted with caution. This finding is based on subjective self-report, which is susceptible to recall and social desirability bias, and the small subgroup sizes preclude statistical inference; differences may also reflect baseline pain severity rather than a true performance advantage of one procedure over the other. Similar trends were observed by Ardebol et al., who found that TSA patients frequently regained the ability to perform high-demand recreational activities, including fishing and shooting sports [18]. For clinical practice, these results underscore the importance of counseling patients that both arthroscopy and arthroplasty can support a return to golf within a year, with skill-specific milestones occurring between five and seven months. Such data can guide rehabilitation protocols, helping patients set realistic goals for progressive return to putting, chipping, and driving.

Although surgical indications for arthroscopy and arthroplasty are distinct and these procedures are not generally interchangeable, the parallel return-to-golf trajectories observed here may help clinicians counsel recreational golfers on realistic recovery expectations following either procedure. These data should not be interpreted as supporting selection between arthroscopy and arthroplasty on the basis of golf-specific outcomes alone, as that decision remains driven by underlying pathology. Future research should explore long-term performance metrics, durability of repair versus prosthesis, and individualized rehabilitation strategies, as well as validate skill-specific return timelines, incorporate objective measures of golf performance, and compare outcomes across different surgical techniques and activity levels to optimize return-to-play protocols.

Limitations

There are several limitations to the current study. Due to the small sample size, a multivariate analysis considering the effects of age, sex, laterality, hand dominance, or preoperative diagnosis was not performed. Second, due to the retrospective nature of the study and the loss to follow-up despite multiple contact attempts, there is a risk of introducing selection bias. Third, there was missing data across several outcome measures, particularly for golf-specific satisfaction and performance items; to address this transparently, a 'not applicable' category was added in Table 5 for any patient who did not provide a response or for whom the question did not apply, allowing all denominators to reflect the full cohort of 71 patients rather than only those with complete responses. These gaps stem from the retrospective, survey-based nature of data collection, in which patients occasionally elected not to respond to specific items or could not be reached for clarification despite repeated contact attempts. Fourth, at this point, there is no clear index for measuring return-to-golf outcomes, and its absence means performance outcomes rely on patient self-report, which is prone to both recall and social desirability bias. Fifth, although all included patients were classified as recreational golfers, there is no standardized definition of 'recreational' play; variability in pre-injury frequency, skill level, handicap, and competitive intensity among participants may influence both subjective satisfaction and reported performance outcomes. Sixth, the mean follow-up duration was approximately 18 to 19 months in both cohorts, which is below the 24-month threshold conventionally used to assess the durability of shoulder surgery outcomes; longer-term data are particularly important for arthroplasty patients, given the relevance of implant longevity. Although all patients met the predefined minimum follow-up of 12 months, sufficient to capture the primary endpoint of return to golf within one year, the durability of these gains over longer time horizons remains unknown. Seventh, the generalizability of these findings may be limited due to the surgeries being performed by a single high-volume surgeon at a single institution. Lastly, there is an increased risk of a type 2 error due to the small sample size used. Additional comparison studies with extended follow-up periods and a more objective measure of golf ability, such as the handicap index, are needed to reach more definitive conclusions.

## Conclusions

In this comparative study, both shoulder arthroscopy and shoulder arthroplasty resulted in substantial improvements in ROM and PROMs among recreational golfers. The majority of patients returned to golf within one year, with high satisfaction and perceived performance. No statistically significant differences were observed in return timelines, golf-specific milestones, or postoperative functional scores between the groups. These findings support the use of either surgical approach to enable active patients to resume recreational sports. Clinicians may implement this information to counsel patients on realistic recovery expectations and to emphasize that, despite differing indications, the surgical approach does not appear to limit return-to-golf potential.
